# Libel Suit against the Editor of the American Journal of Insanity

**Published:** 1878-11

**Authors:** 


### Editorial.

### Libel Suit against the Editor of the American Journal of Insanity.

From the *Buffalo Express* of October 9th, we learn that Dr. WILLIAM A. HAMMOND, of New York, has sued Dr. JOHN P. GRAY, Editor-in-chief of the *American Journal of Insanity* for libel, based on the publication, in the July number of the *Journal of Insanity*, of a paper read at the meeting of Superintendents of American Institutions for the Insane, in May last, by Dr. EUGENE GRISSOM, Superintendent of the State Insane Asylum of North Carolina. Our Editorial brother has our sincere sympathy for this attempt to make him responsible for another man's utterances. We do not see however, how the Doctor is to be held. Dr. Grissom's paper was read at a public meeting in Washington, and a large portion of it published in a daily paper of that city, and its publication acknowledged and replied to by Dr. Hammond in an "Open Letter" issued nearly two months prior to the publication of the *Journal of Insanity.* We learn, furthermore, by referring to the *Journal* that the article complained of was published as part of the proceedings of the Association of Superintendents, without Editorial note or comment.

We have refrained thus far from noticing the pamphlet warfare that has been carried on between Doctors Grissom and Hammond, but we cannot allow the opportunity to pass without protesting against the undignified and in many respects the unprofessional attitude assumed by both parties in the controversy. Dr. Grissom's paper is a severe arraignment of Dr. Hammond's professional career as an author, practitioner, and expert in Courts of Law. We cannot but regret that he did not assume a more dignified tone in his paper, for if true, the charges made, carry enough disgrace without indulging in such epithets as, "Cardiff Giant," "a moral monster whose bowels are but bags of gold," the Cagliostro of to-day, even with his wondrous armory of drugs and stage properties." These are not expressions which should be found in a calm dignified and straight-forward arraignment for unprofessional and dishonest practices."

Dr. Hammond in his "Open Letters" is, however, even more undignified and injudicious, in his use of terms. Instead of boldly denying the charges made by Dr. Grissom, and defying their author to the proof, he indulges in threats of personal violence by saying that had he been present when the paper was read he might have been persuaded to horse-whip the author, and then, in what the *Express* calls "one of the most masterly pieces of satirical writing in the English tongue," attempts to prove Dr. Grissom laboring under mental aberration. We cannot agree with the *Express* in terming this masterly satire, and while holding ourselves wholly neutral upon a question still *sub judice*, cannot but express in most strenuous language our abhorrence of the vituperative abuse heaped upon his opponent by Dr. Hammond. It savors more of the Kearney style of argument than anything we can now call to mind, and cannot but revert with damaging effect upon its author. No provocation should have made Dr. Hammond forget what is due the profession in whose ranks he has held such high position. To call a man a "cacaphonous hypocritical, ranting, howling bigot," to say of him; "a distempered and snarling cur has nobler mental and moral qualities than you; the vibrio that wriggles in decomposing filth is higher in the scale of existence; the foul bird that defecates in his own nest is less odious; the wretch who, actuated by perverted instinct, revels in nastiness and abominations is not so execrable; the monster who insults the mother who bore him is more entitled to human sympathy," is to indulge in language which only disgraces the writer and for quoting which we almost feel inclined to beg our readers' pardon.

In his reply to this Dr. Grissom very properly assumes a more dignified tone, he very plainly intimates that the suit for libel should have been brought against him in North Carolina, and offers to give ample bond to secure the payment of all damages an honest jury may award.

